# Type II Collagen Induces Peripheral Tolerance in BALB/c Mice via the Generation of CD8^+^ T Regulatory Cells

**DOI:** 10.1371/journal.pone.0048635

**Published:** 2012-11-02

**Authors:** Shukkur M. Farooq, Hossam M. Ashour

**Affiliations:** 1 Department of Pharmacy Practice, Eugene Applebaum College of Pharmacy and Health Sciences, Wayne State University, Detroit, Michigan, United States of America; 2 Department of Microbiology and Immunology, Faculty of Pharmacy, Cairo University, Cairo, Egypt; The University of Adelaide, Australia

## Abstract

Antigens introduced into the anterior chamber (AC) of the eye induce a potent form of antigen-specific peripheral immune tolerance termed AC-associated immune deviation (ACAID), which prevents inflammatory immune responses and is characterized by impaired delayed-type hypersensitivity (DTH) responses. Type-II collagen (CII) is a fibrillar protein expressed exclusively in cartilage tissues. Although of its clinical relevance to Rheumatoid arthritis, aging, and osteoarthritis, there have been no studies to date to test if CII has the ability to induce ACAID. We hypothesized that ACAID could be generated via AC injection of CII in BALB/c mice. Using a DTH assay, the hypothesis was supported and led to another hypothesis that CII is capable of inducing specific immune tolerance via CD8^+^ T regulatory cells (Tregs). Thus, we performed functional local adoptive transfer (LAT) assays to examine the regulatory roles of spleen cells, T cells, and CD8^+^ T cells in the specific immune regulation induced by CII injection into the AC. Results indicated that CII induced ACAID when injected into the AC. Spleen cells of mice injected with CII in the AC significantly suppressed DTH responses. The T cell compartment of the spleen was capable of expressing this suppression. CD8^+^ Tregs could solely express this CII-driven suppression and even exerted more noticeable suppression than spleen cells or splenic T cells. This study suggests a crucial role for CD8^+^ Tregs in mediating CII-driven ACAID-mediated immune tolerance. This could have therapeutic implications in Rheumatoid arthritis, aging, osteoarthritis, and other diseases in which CII is involved.

## Introduction

Antigens introduced into the anterior chamber (AC) of the eye induce a potent form of antigen-specific peripheral immune tolerance termed AC-associated immune deviation (ACAID), which prevents inflammation and related deleterious peripheral immune responses [1]–[3]. ACAID is maintained by antigen-specific regulatory T cells (Tregs) [1], [2]. ACAID is functional in mice, primates, and humans [4]. The hallmarks of ACAID include antigen-specific suppression of systemic Th1 immune responses and impaired antigen-specific delayed type hypersensitivity (DTH) responses [5]–[7]. ACAID was also shown to induce antigen-specific inhibition of Th2 mediated pulmonary pathology [8]. Researchers have established that injection of the antigen in the AC is different from intravenous or mucosal antigen delivery, and that the inhibition of cytotoxic T-lymphocyte responses via the AC route in antigen-injected mice was significantly greater [9]. In ACAID, resident ocular tissue F4/80^+^ antigen presenting cells (APCs) process antigens entering into the AC, internalize the antigen before gaining access into the bloodstream and eventually into the spleen [10], [11]. These ocular APCs interact with marginal zone B cells, γδ T cells, and NK T-cells in the spleen in order to induce the differentiation of CD4^+^ afferent Tregs and CD8^+^ efferent Tregs [7], [10], [12], [13]. CD8^+^ Tregs have been shown to have major suppressive roles in autoimmune disease [14].

Type II collagen (CII) is a fibrillar protein expressed exclusively in cartilage tissues (joint articular). Destruction of CII in the articular cartilage could culminate in the loss of joint functions and induce debilitating pain. A study correlated its degeneration to aging and osteoarthritis [15]. Although of the importance and clinical relevance of CII to Rheumatoid arthritis [16], aging, and osteoarthritis, there have been no studies to date to test if CII has the ability to induce specific immune tolerance mediated through ACAID. Thus, we asked the question if ACAID-induced peripheral tolerance could be generated via AC injection of CII in the Th2-skewed BALB/c mice. Here, we show that this form of immune tolerance induction is possible. This led us to the hypothesis that CII is capable of inducing specific immune tolerance in BALB/c mice via CD8^+^ Tregs. Thus, we performed functional assays for the roles of whole spleen cells, T cells, and CD8^+^ T cells in specific immune regulation induced by CII injection into the AC of the eye.

## Materials and Methods

### Mice

BALB/c mice (6–8 weeks of age) were purchased from Jackson Laboratories (Bar Harbor, ME). All animals were maintained in the Eugene Applebaum College of Pharmacy and Health Science Animal Care Facility following the guidelines of the Institutional Animal and Care Use Committee (IACUC), Wayne State University.

### ACAID Induction

ACAID was induced in BALB/c mice using a Hamilton automatic dispensing apparatus (Hamilton, Whittier, CA) as described in our previous publications [7], [10]. BALB/c mice were anesthetized by isoflurane anesthesia (2–3% isoflurane with oxygen supply). About 50–100 µg of CII in PBS (Phosphate-Buffered Saline) was injected (in 5 µl; Sigma, St. Louis, MO) into the AC of the eye. Mice receiving PBS alone via intracameral injection (in the AC of the eye) were used as controls. On day 7, mice immunization was performed by s.c. injection of 250 µg of CII (Sigma-Aldrich). CII was emulsified 1∶1 in complete Freund’s adjuvant (CFA; Sigma-Aldrich). Each animal received 200 µl of a CII/CFA emulsion. On day 14 post AC injection of CII, either a DTH assay or a LAT assay was performed as explained below.

### DTH Assay

On day 14 post AC injection of CII (after 7 days of s.c. immunization of CII/CFA), a DTH assay was performed to measure DTH as discussed in our previous publications [7], [10], [17]. About 500 µg of CII in 20 µl was injected intradermally into the left ear pinnae, and 20 µl PBS alone was injected into the right ear pinnae as an internal control. Both ears were measured 24 h later using a Mitutoyo engineer's micrometer (Japan), and the difference in ear swelling was used as a measure of DTH. The ear swelling measurements were done before and 24 h after CII injection using the following equation: specific ear swelling = (24 h measurement − 0 h measurement) for left ear − (24 h measurement − 0 h measurement) for right ear. There were five animals per group.

### Local Adoptive Transfer (LAT) Assay

This functional assay was performed to test for regulatory cells in ACAID. On day 14 post AC injection of CII (after 7 days of s.c. immunization of CII/CFA), spleens were removed, diced, and expressed through a 40 µm Nylon mesh. All isolated spleen cells were made into single-cell suspensions. Spleen cells containing putative regulatory cells were washed twice and used in a Local Adoptive Transfer (LAT) assay. Details of the LAT assay have been described in some of our previous publications [7], [10]. The *in vivo* generated putative regulatory cells (1×10^6^ cells in 10 µl) together with immune spleen cells (1×10^6^ cells in 10 µl; isolated from s.c. immunized donors) and 500 µg CII were injected into left ear pinna of a naïve mice through intradermal injection (in a total volume of 20 µl), whereas the same volume of PBS alone was injected into the right ear pinna as an internal control. The presence of regulatory cells was assessed by inhibition of ear-swelling responses induced by immune spleen cells after 24 and 48 hrs. In each LAT experiment, the ear pinna of mice in the positive control group were injected with naïve spleen cells plus CII/CFA-immunized (s.c.) effector spleen cells along with the CII antigen (500 µg). Two negative control mouse groups were included in each LAT experiment, one that received 2×10^6^ naive spleen cells (no immune effector cells from previously immunized mice) along with CII (500 µg), and another negative control group of mice that received CII alone (no cells). Positive and negative control groups of mice were injected with PBS alone in the right pinna as an internal control. There were five animals per group.

### Cell Separation by Immunomagnetic Beads

In order to isolate whole T cells or CD8^+^ T cells from spleen, spleen cells of AC-injected mice were incubated with either the CD90.2 (Thy-1.2) or CD8 microBeads (10 µl per 10^7^ cells) (Miltenyi Biotec Inc., CA, USA) for 15 mins at 4–8°C. The cells were subsequently washed twice, and the cell suspension was loaded on a LS MACS column placed in the magnetic field of a MACS separator. The cells were washed 3 times with 3 ml of buffer (PBS containing 0.5% BSA and 2 mm EDTA) and the magnetically labeled T cells or CD8^+^ cells are retained in the column then cells are eluted and resuspended in PBS for injection. The mouse CD90.2 alloantigen is a pan-T cell marker that is expressed on peripheral T cells in BALB/c mice.

### Statistics

The Student’s t test was used to evaluate the significance of experiments. Data were expressed as mean ± SD for all experimental measurements.

## Results

### Injection of CII via the AC Triggers ACAID in BALB/c Mice (DTH Assay)

**Figure 1:**
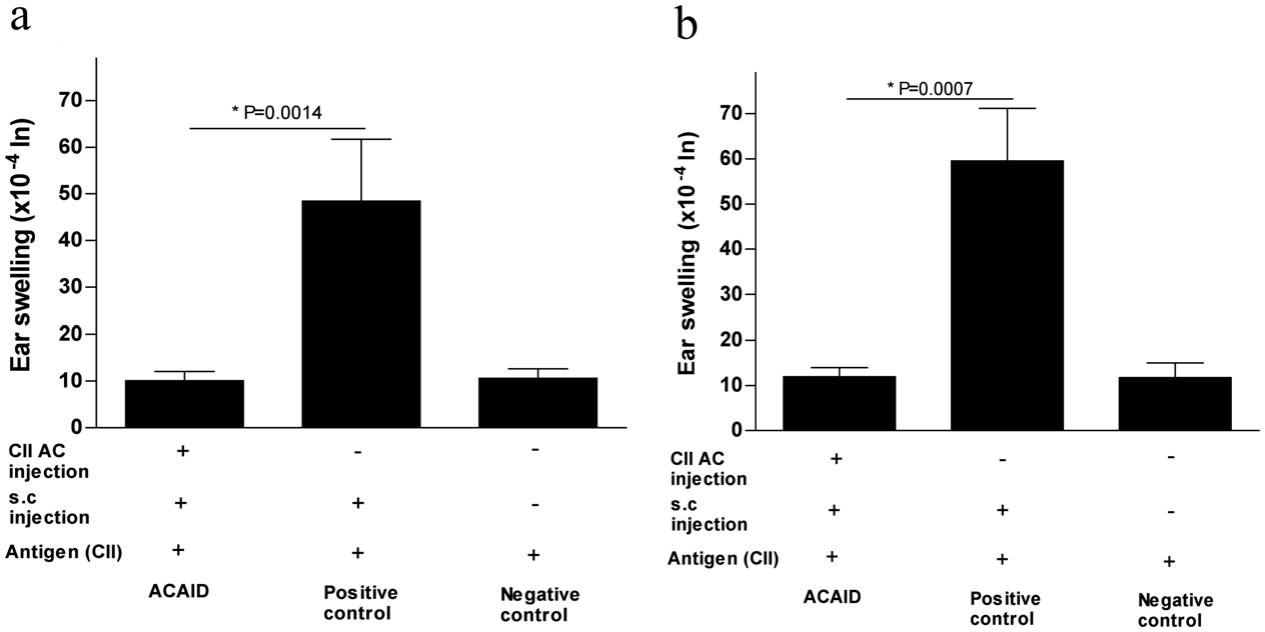
AC injection of CII induced ACAID in BALB/c mice via the suppression of DTH **(DTH assay).** A DTH assay was performed as described in the materials and methods section. To induce ACAID, mice received an AC injection of CII followed by immunization (s.c.) on day 7 with CII/CFA. On day 14, mice were challenged with CII (500 µg in 20 µl) intradermally in the left ear pinna, and 20 µl PBS alone was injected into the right ear pinna as an internal control. Induction of ACAID was confirmed by inhibition of ear-swelling responses after 24 hr (panel ‘a’) and 48 hr (panel ‘b’). The positive control mice were immunized s.c. with CII/CFA on day 7 and with CII on day 14 whereas the negative control mice only received the day 14 intradermal injection of CII.

In order to test whether CII injected into the AC has the ability to induce ACAID in BALB/c mice, we performed a DTH assay. In brief, mice were inoculated in the AC with CII, and then immunized (s.c.) after 7 days with CII/CFA. The animals were subsequently ear-challenged with CII on day 14. Results in figure 1 indicated that the DTH ear swelling response was significantly reduced in mice that received CII in the AC when compared to positive control mice that did not receive the AC injection of CII. Thus, ACAID was successfully induced as a result of CII injection into the AC.

### LAT-based Approaches

DTH inhibition is a hallmark of ACAID at the efferent arm of the immune response [5], [18]–[21]. The LAT assay is one of the most commonly used assays in ACAID research in order to prove the generation of regulatory cells in spleen cells of AC-injected animals. Three LAT assay-based approaches were used to ask different questions regarding the presence of CII-specific regulatory cells. In all three approaches, CII was injected into the AC of BALB/c mice to induce ACAID. Seven days after the AC injection, mice were subcutaneously immunized with CII/CFA. On day 14, mice were sacrificed and spleen cell populations were tested for regulatory functions by a LAT assay, in which both potential efferent suppressor cells and immune spleen cells (from mice previously immunized with CII) were injected into the pinnae of a naive mouse together with the CII antigen. Reduction of ear swelling responses induced by immune spleen cells are indicative of the presence of regulatory spleen cells.

### *In vivo*-generated Spleen Cells (as a Result of CII Injected in the AC) have the Ability to Regulate CII-induced DTH Responses (LAT Assay)

**Figure 2:**
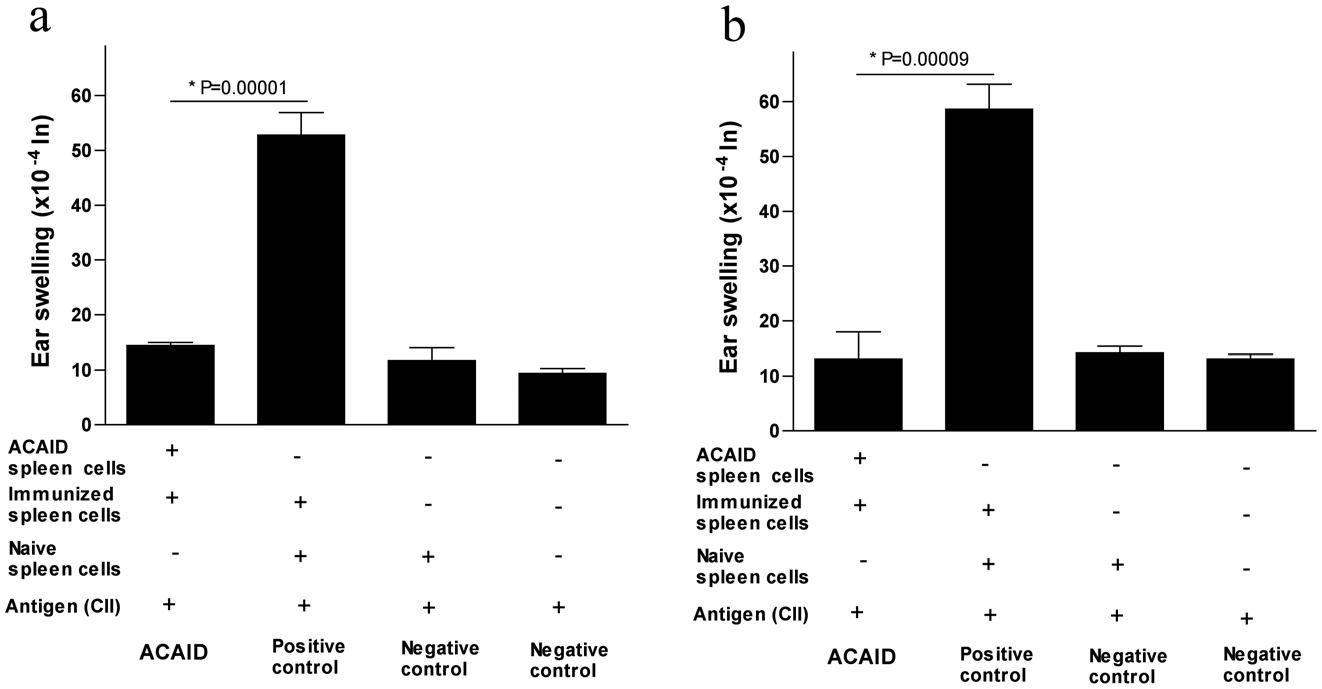
*In vivo*-generated spleen cells (as a result of CII injected in the AC) inhibit CII-induced DTH responses in BALB/c mice (LAT assay). A LAT assay was performed as described in the materials and methods section. Seven days after AC injection, mice were s.c. immunized with CII/CFA. On day 14, putative regulatory spleen cells were isolated and mixed with immune spleen cells (isolated from s.c. immunized mice) and 500 µg CII before injection into the left ear pinna of naïve mice. Positive control mice received immune and naïve spleen cells along with CII. Two negative controls were included; one received only naïve spleen cells along with CII and the other received only CII. The regulatory activity of spleen cells was confirmed by inhibition of ear swelling responses induced by immune spleen cells after 24 hr (panel ‘a’) and 48 hr (panel ‘b’).

In the first approach, whole spleen cell populations were used in the LAT assay. The question is whether spleen cells from CII-injected mice (injected in the AC) display regulatory activities when mixed with immune spleen cells and CII, a possibility that is suggested by our findings in Fig. 1. Spleens were harvested from mice injected with CII in the AC followed by the CII/CFA s.c. immunization in addition to spleens isolated from control naive mice and spleens isolated from CII/CFA-immunized (s.c.) mice. Approximately 1×10^6^ spleen cells of the CII-injected mice (injected in the AC) were combined with an equal number of immune spleen cells (containing effector cell populations) from CII-immunized (s.c) mice along with the CII antigen (500 µg). The total suspension was injected directly into the left ear pinna of naïve mice. The putative regulatory role of spleen cells (isolated from AC-injected mice) was assessed by monitoring the reduction in ear swelling responses induced by immune spleen cells after 24 and 48 hrs as compared to controls lacking the putative regulatory spleen cells. Results indicated that mice in the positive control group developed substantive ear swelling responses, indicative of the inability to suppress or regulate DTH responses of primed immune effector cells after 24 and 48 hrs (Figure 2). Conversely, spleen cells from CII-injected mice (injected in the AC) showed significant (24 hr, P = 0.00001; 48 hr, P = 0.00009) suppression of ear swelling responses as compared to the positive controls (Figure 2). Both negative control groups showed negative ear swelling responses as expected (Figure 2). Thus, ear swelling (DTH) responses induced by primed immune effector cells were directly inhibited by the whole spleen cell population from mice injected in the AC with CII. The reason could be the presence of regulatory T cells within the T cell compartment of this spleen cell population. Thus, we decided to isolate the T cell compartment and test it separately for its regulatory functions.

### *In vivo-*generated Splenic T cells (as a Result of CII Injected in the AC) have the Ability to Regulate CII-induced DTH Responses (LAT Assay)

**Figure 3:**
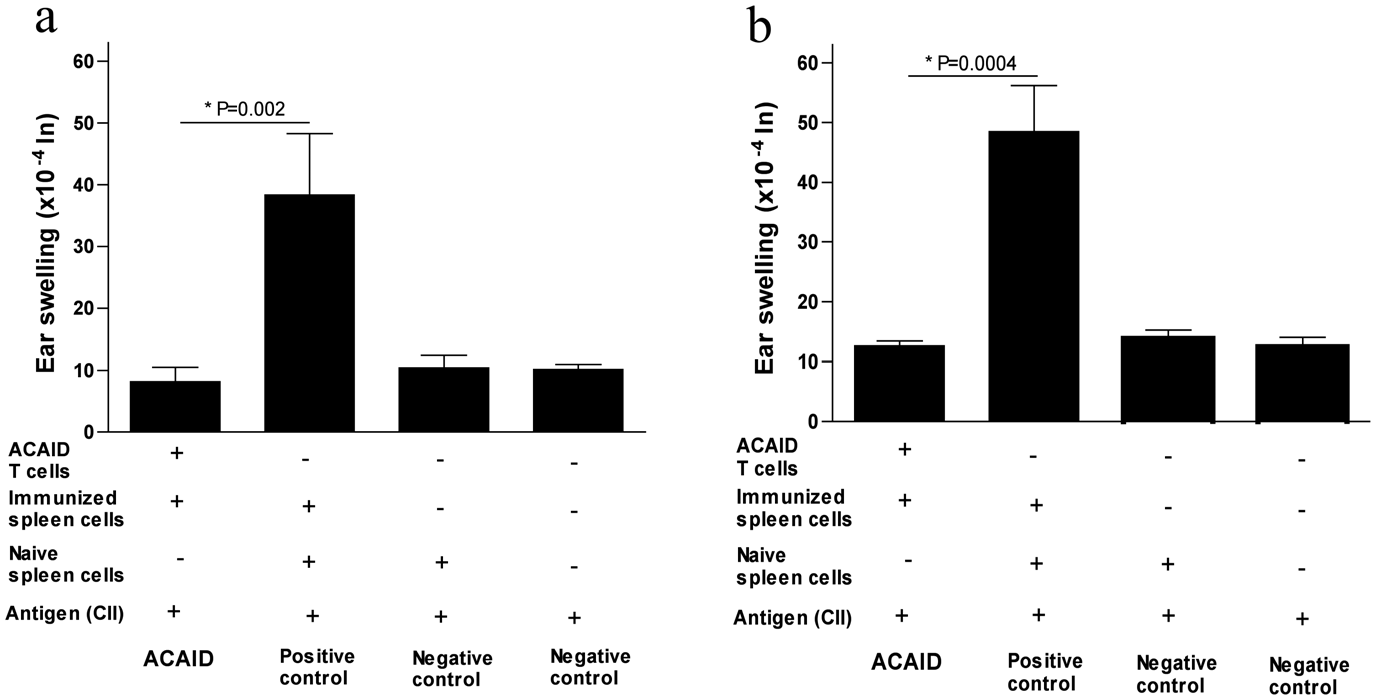
*In vivo*-generated splenic T cells (as a result of CII injected in the AC) inhibit CII-induced DTH responses in BALB/c mice (LAT assay). A LAT assay was performed as described in the materials and methods section. Seven days after AC injection, mice were s.c. immunized with CII/CFA. On day 14, putative regulatory splenic T cells were isolated (CD90.2 microBeads) and mixed with immune spleen cells (isolated from s.c. immunized mice) and 500 µg CII before injection into the left ear pinna of naïve mice. Positive control mice received immune and naïve spleen cells along with CII. Two negative controls were included; one received only naïve spleen cells along with CII and the other received only CII. The regulatory activity of splenic T cells was confirmed by inhibition of ear swelling responses induced by immune spleen cells after 24 hr (panel ‘a’) and 48 hr (panel ‘b’).

In the second approach, *in vivo*-generated splenic T cells were used in the LAT assay instead of the whole spleen cell population used in Fig. 2. The question is whether the T cell compartment in the spleen of CII-injected mice (injected in the AC) displays regulatory activities when mixed with immune spleen cells, a possibility that is suggested by our findings in Fig. 2. Using the CD90.2 immunomagnetic microBeads, the splenic T cell population was isolated from mice injected with CII in the AC followed by the CII/CFA s.c. immunization in addition to splenic cells isolated from control naive mice and splenic cells isolated from CII/CFA-immunized (s.c.) mice. A LAT assay was performed as discussed before. Results indicated that mice in the positive control group developed substantive ear swelling responses, indicative of the inability to suppress or regulate DTH responses of primed immune effector cells after 24 and 48 hrs (Figure 3). Conversely, splenic T cells from CII-injected mice (injected in the AC) showed significant (24 hr, P = 0.002; 48 hr, P = 0.0004) suppression of ear swelling responses as compared to the positive controls (Figure 3). Both negative control groups showed negative ear swelling responses as expected (Figure 3). Thus, ear swelling (DTH) responses induced by primed immune effector cells were directly inhibited by the splenic T cell population from ACAID-induced mice. The non-T cell population did not show any appreciable regulation (data not shown). This shows that the T cell population in the spleen was the regulatory population that suppressed DTH ear swelling responses. Given that efferent CD8^+^ ACAID Tregs mediated DTH inhibition in other ACAID models [5], [18]–[21], we hypothesized that within the whole splenic T cell population, CD8^+^ efferent Tregs solely regulate DTH responses and induce CII-specific immune tolerance.

### *In vivo*-generated Splenic CD8^+^ T cells (as a Result of CII Injected in the AC) have the Ability to Regulate CII-induced DTH Responses (LAT Assay)

**Figure 4:**
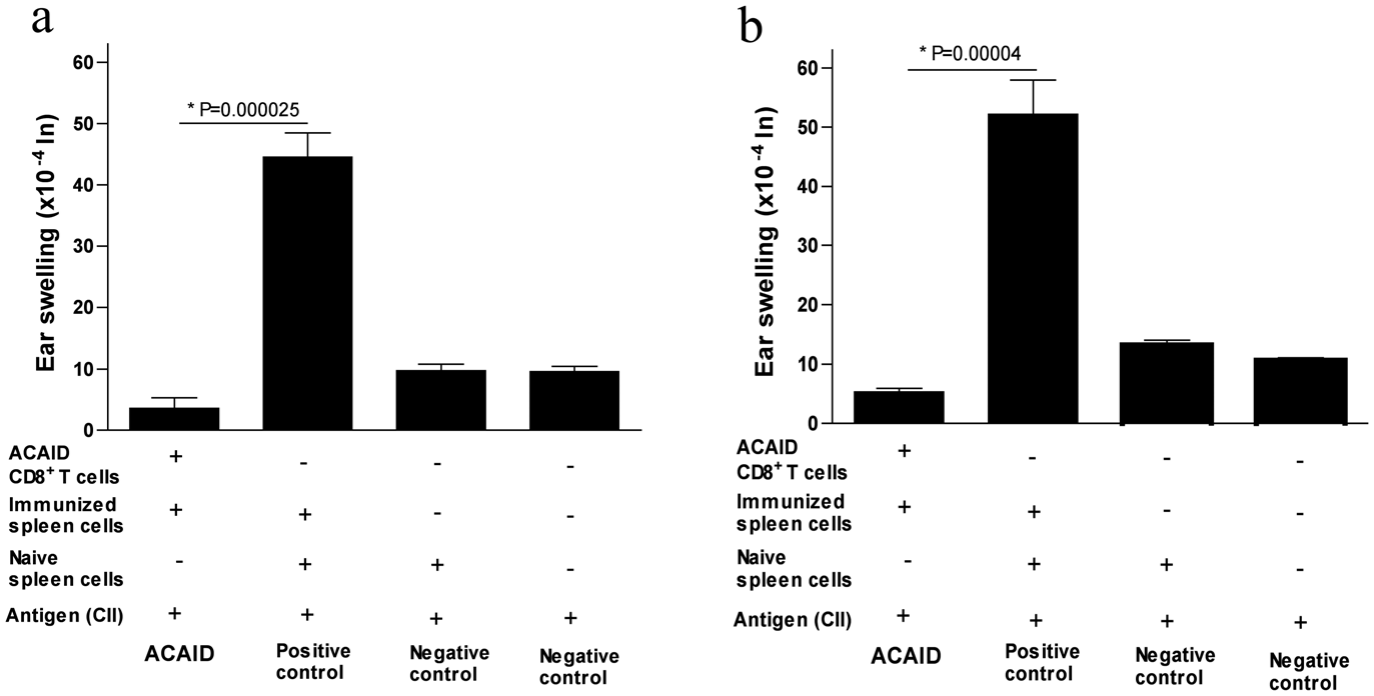
*In vivo*-generated splenic CD8^+^ T cells (as a result of CII injected in the AC) inhibit CII-induced DTH responses in BALB/c mice (LAT assay). A LAT assay was performed as described in the materials and methods section. Seven days after AC injection, mice were s.c. immunized with CII/CFA. On day 14, putative regulatory splenic CD8^+^ T cells were isolated (CD8^+^ microBeads) and mixed with immune spleen cells (isolated from s.c. immunized mice) and 500 µg CII before injection into the left ear pinna of naïve mice. Positive control mice received immune and naïve spleen cells along with CII. Two negative controls were included; one received only naïve spleen cells along with CII and the other received only CII. The regulatory activity of splenic CD8^+^ T cells was confirmed by inhibition of ear swelling responses induced by immune spleen cells after 24 hr (panel ‘a’) and 48 hr (panel ‘b’).

Third approach: In order to test our hypothesis that CD8^+^ efferent Tregs solely regulate CII-specific DTH responses, we isolated the CD8^+^ T cells population from ACAID-induced mice spleens using the CD8 immunomagnetic microBeads and a LAT assay was performed. In contrast to the positive control group in which mice developed substantive ear swelling responses, the ear swelling responses were dramatically diminished when splenic CD8^+^ T cells from CII-injected mice (injected in the AC) were used (24 hr, P = 0.000025; 48 hr, P = 0.00004) (Figure 4). The non- CD8^+^ T cell population did not show any appreciable regulation (data not shown). Interestingly, suppression was noticeably higher when using splenic CD8^+^ T cells (Figure 4) than when using the whole splenic T cell population (Fig. 3).

## Discussion

Immune tolerance induced by antigen entry to an immune privileged site, such as the eye, serves to protect from harmful immune responses. Following antigenic entry into the eye, bone marrow-derived F4/80^+^ APCs of the iris and ciliary body pick the antigen before migrating into the systemic circulation and homing to the spleen where they develop antigen-specific tolerance through the generation of Tregs [10], [11]. The F4/80^+^ molecule has a key role in the differentiation of antigen-specific CD8^+^ Tregs via direct interaction of CD1d^+^ APC with NK T cells [22]. Other studies emphasized the importance of afferent (CD4^+^) Tregs and efferent (CD8^+^) Tregs in the induction of ACAID-mediated immune tolerance [22]–[24]. The efferent arm of immune suppression is crucial in inducing ACAID [25]. In other words, the expression of immune regulation via antigen-specific efferent CD8^+^ Tregs is a major step in the mechanism of ACAID [8], [20], [26].

This study is the first to demonstrate that ACAID could be induced by injecting the CII protein into the AC of the eye. Furthermore, purified CII-driven splenic CD8^+^ T cells were capable of inhibiting DTH responses and were thus proven to be major players in this CII-induced immuno-regulatory response. We previously demonstrated that splenic B cells played critical roles in the generation of both CD4^+^ and CD8^+^ Tregs [10]. We also showed that γδ T cells played a critical role in generating ACAID Tregs [7]. Others have reported that NK T cells, recruited to the spleen via the CXC chemokine ligand 2 (MIP-2; macrophage inhibitory protein-2), were required for the generation of ACAID Tregs [27].

Several studies emphasized potential therapeutic roles for CD4^+^ and CD8^+^ Tregs in autoimmune diseases [28] (such as experimental autoimmune encephalomyelitis [29], [30], Rheumatoid arthritis [31]–[33], lupus [34], and diabetes mellitus type 1 [35]), pulmonary inflammation [8], transplantation [36], and inflammatory bowel disease [37]. Given that antigen-specific inhibitory CD8^+^ Tregs could be useful as potential immunotherapeutic tools in the context of autoimmune diseases, it remains to be demonstrated whether CII protein will drive similar *in vivo* immuno-regulatory responses when tested in the context of an autoimmune disease animal model. Interestingly, suppression with CII-induced CD8^+^ Tregs was noticeably higher than when using splenic T cells or spleen cells in CII-driven ACAID (Figures 2, 3 and 4).

CD8^+^ Tregs are being extensively investigated in multiple sclerosis (MS) and are believed to exert their regulatory effects that could dominate the effects of self-reactive CD4^+^ T cells in MS [38]. In Rheumatoid arthritis, a subset of *in vitro*-generated CD8^+^ Tregs exhibited immuno-regulatory roles, tolerized APCs, inhibited the priming of naïve CD4^+^ T cells, suppressed memory CD4^+^ T cells, and prevented the onset of the anti-inflammatory cascade in synovial lesions [33]. Other studies elucidated the role of CD8^+^ Tregs in Type 1 Diabetes and showed a regulatory activity of CD8^+^ Tregs on autologous, antigen-reactive CD4^+^ T cells in a cell contact-dependent manner [35]. Sugita and colleagues detected TGF-β-producing CD8^+^ Tregs in iris pigment epithelial cells that exhibited a regulatory phenotype, acquired regulatory functions, and were capable of inhibiting bystander effector T cells in the AC [39]. The regulatory subpopulations of the CII-induced CD8^+^ Tregs in our CII-induced ACAID model remain to be elucidated. In other systems, CD8^+^ Tregs were shown to express CD56^+^ [33], CD25^+^ [35], [38], [39], CTLA [35], and FoxP3 [35], [38], [39].

To conclude, we have established that CII could induce ACAID-mediated immune tolerance when injected into the AC of the eye and showed that spleen cells of mice injected with CII in the AC of the eye significantly suppressed DTH responses as compared to control mice. We showed that the T cell compartment of the spleen was capable of expressing this suppression. We finally showed the CD8^+^ Tregs could solely mediate this CII-driven suppression and even exert more significant suppression than spleen cells or splenic T cells. It is noteworthy that splenic CD8^+^ Tregs could have been induced by the presentation of intracameral antigen [27], [40] or may have been converted from CD8^+^ effector T cells to the regulatory phenotype. This study suggests a crucial role for CD8^+^ Tregs in mediating CII-driven ACAID-mediated immune tolerance. This could have therapeutic applications in the context of Rheumatoid arthritis, aging, osteoarthritis, and other diseases in which CII is involved.
